# Epidemiology of moderately severe and severe non-proliferative diabetic retinopathy in South West England

**DOI:** 10.1038/s41433-021-01471-8

**Published:** 2021-03-10

**Authors:** Clareece R. Nevill, Irene M. Stratton, Sonia S. Maruti, Elvira L. Massó-González, Sobha Sivaprasad, Clare Bailey, Michael Ehrlich, Victor Chong, Peter H. Scanlon

**Affiliations:** 1 Gloucestershire Retinal Research Group, Cheltenham General Hospital, Cheltenham, UK; 2 Boehringer Ingelheim Pharmaceuticals Incorporated, Fremont, CA USA; 3 Boehringer Ingelheim International GmBH, Ingelheim am Rhein, Germany; 4 Moorfields Eye Hospital, London, UK; 5 Bristol Eye Hospital, Bristol, UK; 6 Nuffield Department of Clinical Neuroscience, University of Oxford, Oxford, UK; 7 University of Gloucestershire, Cheltenham, UK

**Keywords:** Retinal diseases, Epidemiology

## Abstract

**Aims:**

To estimate the incidence of early treatment diabetic retinopathy study (ETDRS) level 47 and 53 and progression to treatment with panretinal photocoagulation (PRP) for proliferative DR (PDR).

**Methods:**

Log-linear regression was used to estimate the incidence of level 47–53 or worse for 33,009 people with diabetes (PWD) in Gloucestershire during 2013–2016 by calendar year and diabetes type, based on the first recording. Progression was analysed in Gloucestershire and Bristol with a parametric survival analysis examining the association of baseline and time-varying demographic and clinical factors on time to PRP after the first recording of level 47–53.

**Results:**

Incidence decreased from 0.57 (95% confidence intervals (CI) 0.48–0.67) per 100 PWD in 2013 to 0.35 (95% CI 0.29–0.43) in 2016 (*p* < 0.001). For progression, 338 eligible PWD from Gloucestershire and 418 from Bristol were followed for a median of 1.4 years; 78 and 83% had Type 2 diabetes and a median (interquartile range) of 15 (10–22) and 17 (11–25) years duration of diagnosed diabetes respectively. Three years from the incident ETDRS 47–53, 18.9% and 17.2% had received PRP respectively. For Gloucestershire, severe IRMA and updated mean HbA_1c_ were associated with an increase in the risk of initiating PRP (hazard ratio 3.14 (95% CI: 1.60–6.15) and 1.21 (95% CI: 1.06–1.38 per 10 mmol/mol) respectively).

**Conclusion:**

This study provides additional understanding of this population and shows that a high proportion of patients with ETDRS levels 47–53 need to be monitored as they are at high risk of progressing to PDR.

## Introduction

In the UK, 6% of the population have a diagnosis of diabetes [1]. Diabetic retinopathy (DR) is a microvascular complication that is a major cause of blindness and vision loss in the working-age group [2]. In the UK, all people with a diagnosis of diabetes registered with a primary care physician are offered annual digital photographic screening by the NHS diabetic eye screening programme (DESP) in their area from the age of 12 years. The local programmes need to meet standards [3] set by the NHS DESP of minimum attendance of 75% per year and 92% over 3 years. The South West of England has a population of 5.3 million people, 600,000 in Gloucestershire and 900,000 in Bristol and the surrounding area. Each area is served by one DESP and one hospital eye service (HES) with 33,000 people with diabetes (PWD) and 44,000 PWD, respectively. This study used retrospective data from the two HESs and the Gloucestershire DESP. The Bristol and Weston DESP changed their software supplier in October 2013 making it impossible to analyse DESP data from the Bristol area.

Moderately severe non-proliferative DR (NPDR) and severe NPDR are classified as early treatment diabetic retinopathy study [4] (ETDRS) level 47 and 53, respectively. They are classified by the presence and severity of venous beading, intraretinal microvascular abnormalities (IRMA), and multiple haemorrhages compared to standard photos—the ETDRS produced standard photos to compare lesions e.g., standard photograph 8A for IRMA and 2A for haemorrhages. The treatment recommended by the UK Royal College of Ophthalmologists [5] for proliferative DR (PDR) is panretinal photocoagulation (PRP) with a few patients treated at level 53 where there is concern about patient compliance [6]. Moderately severe NPDR is not amenable to eye-specific treatment though patients are advised that the risk of progression to sight-threatening retinopathy will be reduced if glycaemic control is optimal. This study aims to characterise the moderately severe and severe NPDR population since this information is currently limited.

## Materials and methods

Gloucestershire DESP (GDESP) has offered annual digital photographic screening to all eligible people in Gloucestershire since 1998. Screening is carried out in healthcare settings including primary care practices and clinics in hospitals. Gloucestershire hospital eye service (HES) clinics are run in the two main hospitals and five other clinics across Gloucestershire. Bristol HES clinics are run in Bristol Eye Hospital and five other clinics across the region.

Patient and public involvement—Gloucestershire has a group of three PWD who we have consulted within the design and writing up phase of this study.

### Study design and study population

A retrospective, observational analysis using data from PWD aged 18 years and older in Gloucestershire and Bristol was conducted between 1 January 2013 and 31 December 2016. The cohort was defined as those in 2012 or later who have not yet reached EDTRS level 47 in their worst eye. The Gloucestershire cohort consisted of patients who had GDESP electronic screening medical records (ESMR—OptoMize from Northgate Ltd., Hemel Hempstead, UK) and HES electronic medical records (EMR—Medisoft Limited, Leeds, UK). The Bristol HES cohort had HES EMR (Medisoft) data.

Data collection pseudonymised data were extracted from the Gloucestershire ESMR and EMR and from the Bristol EMR for attendances between 1 January 2012 and 31 December 2016.

### Statistical methods

#### Primary analysis

Incidence of moderately severe NPDR or worse (ETDRS level ≥47) in 2013–2016.

Patient population analysed: Patients from the Gloucestershire cohort.

The patients at risk had one complete DR prior assessment with DR of ETDRS level <47 in both eyes. Incident cases (numerator) were defined as first-time ETDRS level ≥47 in at least one eye was recorded during 2013–2016. This included those of level 53 and above who had progressed from <47 directly to a higher level. Patients who already received PRP, anti-VEGF, or steroids were excluded.

Incidence estimates were calculated with 95% confidence intervals (CI) over time using log-linear (Poisson) regression models and also analysed by diabetes type.

#### Secondary analyses

Time to initiate PRP treatment among patients with ETDRS 47 or 53 in the Gloucestershire and Bristol cohorts.

Patient population analysed: patients from both centres were included if they had a first recording of moderately severe—severe NPDR diagnosis (ETDRS level 47–53) in 2013–2016 and at least one subsequent follow-up record. Those with a previous record of ETDRS level ≥47 were excluded, as were those who had been treated with PRP, intravitreal injection treatment of VEGF inhibitors or steroids.

Hazard ratios (with 95% CI) were calculated from univariate and multivariate Weibull models (fitted using forward stepwise selection) to evaluate time to initiation of PRP treatment from the first record of DR of ETDRS level 47–53 in the worse eye. Patients were censored at death, moving out of the area, loss to follow-up (those still registered but were last seen over a year before study endpoint), or end of 2016, whichever came first.

The updated HbA_1c_ for an individual at any time point is the weighted mean of all previous HbA_1c_ measures for that patient since the earliest HbA1c assessment (baseline) during the study period, with more weight given to more recent assessments. All secondary analyses were run separately for Gloucestershire and Bristol because of extra data available from the Gloucestershire site e.g., HbA_1c_, ethnicity, and ESMR data.

Statistical analyses were performed using Stata 16. Ethics approval was granted by the NHS Health Research Authority for this study with IRAS project ID: 236309.

## Results

### Primary analysis

In Gloucestershire 33,009 PWD met the inclusion/exclusion criteria for the incidence analysis in 2013–2016. They were aged 67 (56–76) years (median, interquartile range), 57% male, 94% had Type 2 diabetes, with 6 (2–10) years duration of diagnosed diabetes.

For each calendar year, the incidence of ETDRS level ≥47 was estimated (Table 1). Incidence decreased from 0.57 (95% CI: 0.48–0.67) per 100 PWD in 2013 to 0.35 (95% CI: 0.29–0.43) in 2016, with incidence rate ratio (IRR) for calendar year 0.86 (95% CI: 0.79–0.93) *p* < 0.001. When split by diabetes type, the downward trend with time was only found amongst those with T2DM (*p* = 0.587 for trend with time for T1DM). For Gloucester people with T2DM, incidence of ETDRS level ≥47 decreased from 0.47 (95% CI: 0.39–0.57) per 100 PWD in 2013 to 0.26 (95% CI: 0.20–0.33) in 2016 (IRR for calendar year 0.83 (95% CI: 0.76–0.91) *p* < 0.001).

**Table 1:** Incidence of moderately-severe NPDR or worse (ETDRS level ≥47), per 100 Gloucestershire people with diabetes.

Number of Gloucestershire PWD at risk* of developing incident moderately severe NPDR or worse in at least one eye during the respective year			2013	2014	2015	2016	IRR (95% CI) for trend over time (increment of the calendar year)
		Overall±	23,860	25,683	27,175	29,393	
		T1DM	1432	1578	1685	1761	
		T2DM	22,410	24,030	25,394	27,501	
New ETDRS level 47 or worse in at least one eye	Overall	*n*	136	119	119	103	0.86
		Incidence	0.57	0.46	0.44	0.35	(0.79–0.93)
		(95% CI)	(0.48–0.67)	(0.39–0.55)	(0.37–0.52)	(0.29–0.43)	*p* < 0.001
	T1DM	*n*	31	26	30	32	0.96
		Incidence	2.2	1.7	1.8	1.8	(0.81–1.12)
		(95% CI)	(1.5–3.1)	(1.1–2.4)	(1.2–2.6)	(1.3–2.6)	*p* = 0.587
	T2DM	*n*	105	93	89	71	0.83
		Incidence	0.47	0.39	0.35	0.26	(0.76–0.91)
		(95% CI)	(0.39–0.57)	(0.32–0.47)	(0.28–0.43)	(0.20–0.33)	*p* < 0.001

*PWD* people with diabetes, *NPDR* non-proliferative diabetic retinopathy, *T1DM* Type 1 diabetes mellitus, *T2DM* Type 2 diabetes mellitus, *CI* confidence interval;

*Those at risk were those on the GDESP register during the respective year, with at least one assessment that year and where all prior assessments showed DR of ETDRS level <47 in both eyes.

±Includes those with ‘other’ and ‘unknown’ diabetes types.

### Secondary analysis

In the Gloucestershire cohort, 477 were newly diagnosed with level 47 or worse: at the time of first recording, 110 (23.1%) were level 47, 228 (47.8%) level 53 and 139 (29.1%) PDR (level > 61). Among the Bristol cohort, 550 people were newly diagnosed with level ≥47: 227 (41.3%) were level 47, 191 (34.7%) level 53 and 132 (24.0%) PDR (level > 61). Those with PDR at first recording were excluded from the progression analysis. Thus, 756 people (338 from Gloucestershire and 418 from Bristol) met the inclusion criteria for the secondary analysis, with a median follow up time of 1.4 years for both Bristol and Gloucestershire. Baseline characteristics are shown in Table 2.

**Table 2:** Baseline patient characteristics of those in the secondary (survival) analysis; Gloucestershire (*N* = 338) and Bristol (*N* = 418) PWD with incident DR of ETDRS level 47–53 during 2013–2016.

		Gloucestershire (*N* = 338)		Bristol (*N* = 418)	
		*N*	%	*N*	%
Gender	Female	142	42.0	175	41.9
	Male	196	58.0	243	58.1
Ethnicity	Recorded	336	99.4	n/a^a^	
	Caucasian	311	92.6		
	Asian	16	4.8		
	Black	5	1.5		
	Mixed	2	0.6		
	Other	2	0.6		
Diabetes type	Recorded	338	100	390	93.3
	T1DM	74	21.9	65	16.7
	T2DM	264	78.1	325	83.3
HbA_1c_ (mmol/mol)	Recorded	327	96.7	n/a^b^	
	Median (IQR)	68 (56–85)			
	Mean (SD)	71.6 (20.4)			
Time since diagnosis of diabetes (years)	Recorded	338 (100%^c^)		219 (52.4%)	
	Median (IQR)	15 (10–22)		17 (11–25)	
	Mean (SD)	16.2 (9.4)		19.1 (12.2)	
Age (years)	Median (IQR)	63 (52–72)		63 (52–73)	
	Mean (SD)	61.4 (14.8)		61.7 (14.6)	
VA (Log MAR)^d^	Recorded	338 (100%)		417 (99.8%)	
	Median (IQR)	0.1 (0.0–0.2)		0.1 (0.0–0.2)	
	Mean (SD)	0.13 (0.20)		0.11 (0.17)	
Retinopathy grade in the better eye	No DR (ETDRS 10)	0	0.0	4	1.0
	Mild NPDR (ETDRS 20–35)	75	22.2	115	27.5
	Moderate NPDR (ETDRS 43)	142	42.0	117	28.0
	Moderately severe NPDR (ETDRS 47)	48	14.2	105	25.1
	Severe NPDR (ETDRS 53)	73	21.6	77	18.4
	PDR (ETDRS ≥ 61)	PDR at baseline were excluded from the analysis			
Maculopathy in either eye	M0 in both eyes	165	48.8	125	29.9
	M1 in at least one eye	173	51.2	293	70.1
Presence of venous beading in either eye	No VB	203	60.1	277	54.3
	VB in at least one eye	135	39.9	191	45.7
Presence of IRMA in either eye	No IRMA	30	8.9	134	32.1
	IRMA < 8a in at least one eye	112	33.1	179	42.8
	IRMA > 8a in at least one eye	196	58.0	105	25.1
Presence of multiple haemorrhages in either eye	No multiple haemorrhages	251	74.3	187	44.7
	Multiples haemorrhages in at least one eye	87	25.7	231	55.3

Baseline was the time when a participant was first found to have DR of ETDRS level 47–53 in at least one eye.

*T1DM* Type 1 diabetes mellitus, *T2DM* Type 2 diabetes mellitus, *NPDR* non-proliferative diabetic retinopathy, *VB* venous beading, *IRMA* intraretinal microvascular abnormalities, *IQ* inter-quartile range, *VA* visual acuity.

^a^For Bristol, 18.9% had missing ethnicity. Of those with ethnicity recorded, 98.2% were recorded as Caucasian. The research team felt that this was not representative of the true ethnicity distribution in Bristol and so removed ethnicity from Bristol analyses.

^b^HbA_1c_ data was not available for participants from Bristol.

^c^3 Gloucestershire participants had no diabetes diagnosis date available, for those date of DESP registration was used as a proxy.

^d^Not all participants had a VA measure on their EMR of precision 2 decimal places. One Gloucestershire participant had a VA of count fingers, this was converted to 2.0 Log MAR.

Of the 756 participants, 46 (13.6%) from Gloucestershire and 39 (9.3%) from Bristol were subsequently treated for DR with PRP. Of the 671 participants who did not go on to receive PRP treatment for DR:

(a) Gloucestershire cohort—at their last assessment, 6 (2.1%) had untreated PDR, 251 (86.0%) level 47–53, 32 (11.0%) mild NPDR and 3 (1.0%) no DR. The latter three were felt to be unlikely and so were audited by PHS. One was considered an error, the second a vein occlusion (not DR) and the third caused by lesions outside the photographic fields of the second assessment. During follow-up, 24 (7.1%) died, 45 (13.3%) were not seen again in over a year, and 5 (1.5%) moved away.

(b) Bristol cohort—at their last assessment, 7 (1.9%) had untreated PDR, 315 (83.3%) level 47–53, 62 (16.4%) mild NPDR and 1 (0.3%) no DR. The one with no DR was audited by the Bristol Clinical Lead and was considered an error in the original diagnosis. During follow-up, 8 (2.1%) died (although the recording of this was incomplete) and 61 (16.1%) were not seen again in over a year or moved away (specific data unavailable).

**Fig. 1:**
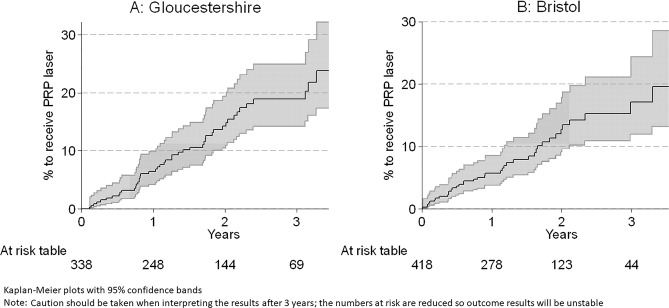
Time to PRP laser from Incident ETDRS level 47–53. Gloucestershire (**A**) and Bristol (**B**).

Kaplan–Meier plots of time to treatment for patients with incident ETDRS level 47–53 (baseline) are shown in Fig. 1. In Gloucestershire, after 1 year 6.4% (95% CI: 4.1–9.9%) and after 3 years 18.9% (95% CI: 14.2–25.0%) had received PRP. In Bristol, after 1 year 5.7% (95% CI: 3.7–.6%) and after 3 years 17.2% (95% CI: 11.9–24.4%) had received PRP. There was no difference in these rates between Gloucestershire and Bristol (*p* = 0.43).

The results from the univariate and multivariate analyses of time to PRP are shown in Tables 3 and 4, the latter finding the following risk factors for requiring PRP: (a) in Gloucestershire those with IRMA > 8a and those with continuously higher HbA_1c_ levels. (b) In Bristol those who were younger, those with VB in either eye, those with DR of ETDRS level 53 in the better eye (at baseline), and those with maculopathy in either eye.

**Table 3:** Risk factors for initiating PRP treatment: univariate analyses.

Baseline factors	Gloucestershire (*N* = 338)		Bristol (*N* = 418)	
	Univariate HR (95% CI)	*p*-value	Univariate HR (95% CI)	*p*-value
Sex: female	1.56 (0.87–2.78)	0.138	1.32 (0.71–2.48)	0.385
Duration of diabetes (per 5 years)^a^	1.00 (0.85–1.16)	0.950	0.87 (0.71–1.06)	0.163
Age (per 5 years)	0.86 (0.78–0.95)	0.003	0.79 (0.71–0.87)	<0.001
Type of diabetes: T1DM^b^	1.27 (0.66–2.44)	0.483	2.14 (1.04–4.42)	0.040
Retinopathy level in the better eye (ref: ETDRS 10–35)^c^				
ETDRS 43	1.23 (0.56–2.71)	0.601	2.13 (0.81–5.61)	0.125
ETDRS 47	1.11 (0.37–3.31)	0.856	1.72 (0.60–4.95)	0.316
ETDRS 53	1.75 (0.74–4.16)	0.205	3.96 (1.49–10.57)	0.006
Ethnicity: Caucasian	0.83 (0.30–2.31)	0.716	n/a	
Time-varying factors				
VB in at least one eye vs. none	1.54 (0.86–2.76)	0.145	2.85 (1.50–5.42)	0.001
IRMA status (ref: no IRMA in both eyes)				
IRMA < 8a in at least one eye	1.51 (0.33–6.92)	0.592	1.58 (0.68–3.65)	0.287
IRMA > 8a in at least one eye	4.90 (1.17–20.52)	0.029	4.21 (1.85–9.57)	0.001
MH in at least one eye vs. none	1.62 (0.86–3.05)	0.132	1.86 (0.99–3.52)	0.055
VA (per 0.1 increment in LogMAR score)	1.05 (0.94–1.17)	0.411	0.94 (0.74–1.19)	0.612
Maculopathy: M1 in either eye	1.14 (0.60–2.16)	0.695	2.04 (1.09–3.83)	0.026
Updated mean HbA_1c_ (per 10 mmol/mol)^d^	1.24 (1.09–1.42)	0.001	n/a	

*DR* diabetic retinopathy, *HR* hazard ratio, *T1DM* Type 1 diabetes mellitus, *VB* venous beading, *IRMA* intraretinal microvascular abnormalities, *MH* multiple haemorrhages, *VA* visual acuity, *PRP* panretinal photocoagulation.

^a^For Bristol, the diabetes diagnosis date was only available for 219 (52.4%) participants, of which 23 received PRP.

^b^For Bristol, diabetes type was known for 390 (93.3%) participants, of which 31 received PRP.

^c^Four patients in Bristol had no DR in their fellow eye; the significant HR for having ETDRS 53 in the fellow eye remained significant when removing those with no DR from the reference group.

^d^For Gloucestershire, the updated mean HbA_1c_ was available was 327 (96.7%) participants, of which 45 received PRP.

**Table 4:** Risk factors for initiating PRP treatment: multivariate analyses.

Gloucestershire^a^		
Risk factor	Multivariate HR (95% CI)	*p*-value
IRMA > 8a in either eye vs. IRMA < 8a or none (time-varying)	3.14 (1.60–6.15)	0.001
Updated mean HbA_1c_ (per 10 mmol/mol, time-varying)	1.21 (1.06–1.38)	0.005
*Bristol*		
Age (per 5 years, time-varying)	0.79 (0.70–0.88)	<0.001
VB in either eye vs. none (time-varying)	2.71 (1.43–5.16)	0.002
ETDRS 53 in fellow eye vs. ETDRS ≤ 47 (at baseline)	2.33 (1.17–4.62)	0.016
M1 in either eye vs. M0 in both (time-varying)	2.02 (1.07–3.81)	0.029

Results are from multivariate Weibull models (through forwarding step-wise selection) for time to PRP treatment for DR after having DR of ETDRS level 47–53 recorded in Gloucestershire and Bristol people.

*DR* diabetic retinopathy, *HR* hazard ratio, *T1DM* Type 1 diabetes mellitus, *VB* venous beading, *IRMA* intraretinal microvascular abnormalities, *MH* multiple haemorrhages, *VA* visual acuity, *PRP* panretinal photocoagulation.

^a^Based on 327 participants as a result of having updated mean HbA_1c_ in the model.

## Discussion

This study aimed to characterise moderately severe and severe NPDR patients since this information is currently limited. Wong et al. [7] reported that detailed reporting of ETDRS levels was only present in 4% of studies reviewed.

Although International Coding Systems [8, 9] do contain DR levels, the EMR system used in the two HESs is unique in that it requires the clinician to fill in a structured assessment form based on lesion identification and the system assigns an ETDRS level.

We were unable to find any studies in the literature that specifically reported on the incidence of levels ≥47 although a number of studies have reported on the incidence of PDR in those who had not had PDR at baseline.

In Gloucestershire, the incidence of ETDRS level ≥47 decreased during the study period. Although there were low numbers of non-attenders they are more likely to have higher levels of retinopathy [10, 11]. It was not possible to estimate incidence rates in the Bristol cohort because of the lack of primary care screening data to determine the denominator.

In 1989, Klein reported the 4-year incidence of PDR [12, 13] was 11% in those diagnosed <30 years of age, and 7% for insulin users and 2% for non-insulin users ≥30 years of age. In 2008, Klein reported the 25-year incidence of PDR [14] in Type 1 diabetes was 42%.

In 2010, Varma [15] reported the 4 years incidence of PDR from the Los Angeles Latino Eye Study of Type 2 diabetes was 5.3%. In 2014 Broe reported [16] the 16 years incidence of PDR in Type 1 diabetes was 31%.

The 4-year incidence of ETDRS level ≥47 was 1.45% (95% CI: 1.32 to 1.58). 94% had Type 2 diabetes with a median age of 67 (IQR 56–76) years. This is lower than the 2% who developed PDR for non-insulin users ≥30 years in the 1989 Klein paper and lower than the 5.3% reported by Varma. The most likely reason for this and the reduction over the period of the study is better glycaemic and blood pressure control.

In the ETDRS study [4], 3 years progression to PDR was 47.6% for level 47 and 71.1% for level 53.

Klein reported [17] the 10 years progression to PDR in those diagnosed <30 years was 82.0% for level 47 and 75.0% for level 53, and for those diagnosed ≥30 years was 80.5% for level 47 and 61.5% for level 53. The UKPDS study [18] reported 6 years progression to PDR requiring PRP was 60 and 90% from levels 47 and 53 in one eye respectively. An EMR study from 19 UK hospitals reported [19] the 3 years progression to proliferative was 16.1% for eyes with level 43 (*n* = 6986), 31.6% for eyes with level 47 (*n* = 1764) and 55.8% for eyes with level 53 (589).

In this study 18.9% of patients in Gloucestershire and 17.2% in Bristol had received PRP within 3 years of developing DR of ETDRS level 47–53 in their worst-affected eye, indicating slower progression in this group than previously reported.

UK Screening studies [20–23] tend to report the incidence of referable retinopathy which is standardised across UK screening programmes as level ≥43 and maculopathy. Hence these studies have limited data on levels 47–53. A screening study [24] in two diabetes clinics in Northern Italy refers at level ≥47 and maculopathy and they reported a 21.1% 10 years referral rate if level 43 was present at first examination.

It is well known that high levels of HbA_1c_ [12–14, 16, 18] are a major risk factor for progression of DR. Klein [14], Wong [7] and Kiore [25] all reported lower rates of DR progression in later time periods which is felt to be due to better control of glycaemia and blood pressure.

We found prominent IRMA (>8A) and HbA_1c_ as the two highest risk factors for progression in the Gloucestershire cohort and that rates of progression are much lower than earlier time periods. This is in agreement with previous literature [4, 26], but this study helps to quantify incidence and progression in the modern era where glycaemic and blood pressure treatment guidelines are tighter. The Bristol multivariate analysis indicated a negative relationship with age and progression; this goes against common knowledge of the disease. Two possible reasons for this result are: (a) the true relationship with age may not fit the model assumptions and so more sophisticated modelling may be needed, or (b) instead of indicating that younger people are more likely to progress, it may be indicating that older people do not survive long enough for the disease to progress this far. The Bristol multivariate analysis was limited by the unavailability of HbA_1c_ data. Hence the Gloucestershire results have been taken as primary to form conclusions and Bristol is supportive, warranting further research. In conclusion, the real-world data in this study is unique in the level of detail of ETDRS levels that are recorded and analysed. It can answer patients’, ophthalmologists’ and DESP managers’ queries related to current clinics that monitor those with moderately severe—severe NPDR (EDTDRS levels 47–53). By looking at data from 2012 to 2016 this study gives recent data demonstrating that a high proportion of patients with ETDRS levels 47–53 progress to PDR requiring further medical interventions (currently PRP). These patients have a currently unmet medical need to slow down progression or to reverse their condition, especially for those with the highest risk of progression with poorly controlled diabetes. However further research is needed to help clinicians identify ways in which the risk of progression can be reduced.

### Summary

#### What was known before

- This study aimed to characterise moderately severe and severe NPDR patients.
- This information is currently limited.
- Detailed reporting of ETDRS levels is only present in 4% of studies.

#### What this study adds

- The real-world data in this study is unique in the level of detail of ETDRS levels that are recorded and analysed.
- The association of baseline and time-varying demographic and clinical factors on time to PRP after the first recording of level 47–53.
